# Meta-signature LncRNAs serve as novel biomarkers for colorectal cancer: integrated bioinformatics analysis, experimental validation and diagnostic evaluation

**DOI:** 10.1038/srep46572

**Published:** 2017-04-13

**Authors:** Meiyu Dai, Xiaoli Chen, Shanying Mo, Jinwan Li, Zhizhuo Huang, Shifeng Huang, Junyi Xu, Baoyu He, Yan Zou, Jingfan Chen, Shengming Dai

**Affiliations:** 1 Medical Science Laboratory, the Fourth Affiliated Hospital of Guangxi Medical University, Liuzhou, Guangxi 545005, China; 2 Department of General Surgery, the Fourth Affiliated Hospital of Guangxi Medical University, Liuzhou, Guangxi 545005, China

## Abstract

The aim of this study is to explore the differentially expressed lncRNAs, which may have potential biological function and diagnostic value in colorectal cancer (CRC). Through integrated data mining, we finally identified nine differentially expressed lncRNAs and their potential mRNA targets. After a series of bioinformatics analyses, we screened significant pathways and GO terms that are related to the up-regulated and down-regulated transcripts respectively. Meanwhile, the nine lncRNAs were validated in 30 paired tissues and cell lines by qRT-PCR and the results were basically consistent with the microarray data. We also tested the nine lncRNAs in the serum of 30 CRC patients matched with the CRC tissue, 30 non-cancer patients and 30 health controls. Finally, we found that BLACAT1 was significant for the diagnosis of CRC. The area under the curve (AUC), sensitivity and specificity were 0.858 (95% CI: 0.765–0.951), 83.3% and 76.7% respectively between CRC patients and health controls. Moreover, BLACAT1 also had distinct value to discriminate CRC from other non-cancer diseases. The results indicated that the differentially expressed lncRNAs and their potential target transcripts could be considered as potential therapeutic targets for CRC patients. Meanwhile, lncRNA BLACAT1 might represent a new supplementary biomarker for the diagnosis of CRC.

Cancer is the biggest threat to human life and now has been a hotspot in academic research around the world. Statistical report indicates that there are 1,665,540 cases of new cancers and 585,720 cancer-related deaths every year in the United States^1^. Among them, colorectal cancer (CRC) is the second most common cancer in female and third in male with the occurrence of over one million new cases and half a million deaths by the global cancer statistics^2^. In China, the incidence of CRC ranks third and its mortality ranks fourth in various cancers^3^. Although considerable progress has been made in medical science, the incidence and mortality of CRC are still increasing year by year^4^. Therefore, it is essential to find novel biomarkers for the early diagnosis and targeted therapy for CRC patients.

Long non-coding RNAs (lncRNAs), following siRNA and microRNA, are another new class of transcripts that have been found pervasively associated with human diseases. LncRNA, with its sequence of longer than 200 bp, has the poly A tail and promoters in structure after splicing^5^. In the different processes of tissue differentiation and development, most lncRNAs have obviously specific expression. Meanwhile, they also have different expression patterns in different parts of the human tissue^6^,^7^. At present, several screening diagnosis methods for CRC are widely used, including digital rectal examination, imaging examination, endoscopy, fecal occult blood examination and tumor biomarkers detection. But they all have limitations in such aspects as high false negative rate, complex operation process, low sensitivity and specificity, and excessive dependence on the clinical phenotype. With the rapid development of chip technology and high-throughput sequencing, the genetic biomarkers in tumor have become increasingly valuable. A growing body of research indicate that lncRNAs play crucial roles in various human diseases, especially in cancers^8^,^9^,^10^,^11^, some of which become classic research hotspots and are studied more comprehensively. For example, lncRNA HOTAIR is a poor prognostic indicator for breast cancer metastasis, pancreatic cancer, CRC and is also related to cell cycle progression in human glioma^12^,^13^. However, individual lncRNA in the diagnosis and prognosis of the disease is lack of specificity, so lncRNA expression signature or profile has been adopted by many researchers^10^,^14^,^15^. In recent years, there are many studies on lncRNAs in CRC. For instance, lncRNA CRNDE, is not only associated with the diagnosis and prognosis of CRC, but also promoted CRC cell proliferation and chemoresistance^16^,^17^. LncRNA H19 was an independent prognostic factor and mediated methotrexate resistance in colorectal cancer through Wnt/β-catenin signal pathway^18^,^19^. However, they are also differential expression genes and play an important role in other cancers like glioma^20^,^21^, ovarian cancer^22^,^23^ and hepatic carcinoma^24^,^25^. Therefore, more novel lncRNAs associated with CRC need to be found and detected in the future application.

The rapid development of modern gene chip technology and bioinformatics analysis makes it possible to identify and study more novel lncRNAs. Many researchers start to search for the significant differential expression genes through the data mining from public databases. Then, the functional enrichment analysis and experimental verification of target genes are carried out to provide reference for clinical target treatment, early diagnosis and prognosis evaluation^26^,^27^,^28^. Some evidence indicate that lncRNAs acting as competing endogenous RNAs (ceRNAs) are involved in a variety of tumor initiation and progression. Then systematic studies on lncRNA-associated ceRNA network also have been performed in various cancers such as ovarian cancer^29^, breast cancer^30^, and gastric cancer^31^. It could provide novel insights to understand the molecular mechanism and functions of lncRNAs in cancers.

In our study, a systematic expression profiling analysis was introduced to identify meta-signature lncRNAs between the CRC tissues and adjacent normal tissues. Meanwhile, these selected lncRNAs were also validated in tissues and cell lines. Then bioinformatics analysis was adopted to further study the target genes and functional annotation of the differentially expressed lncRNAs, which might contribute to the research on targeted therapy of CRC. Finally, we also detected the expression levels of lncRNAs in serum among CRC patients, non-cancer patients and health controls to find out novel biomarkers for the diagnosis and differentiation of CRC.

## Results

### Differentially expressed lncRNAs and mRNAs in the microarray datasets

In our study, a total of eight datasets were included for comprehensive analysis (GSE77199, GSE76855, GSE32323, GSE62321, GSE8671, GSE20842, GSE39582, and GSE21815). After data preprocessing, we finally found 21,439 mRNAs and 1,299 lncRNAs in GSE77199 dataset, 12,319 mRNAs and 2,367 lncRNAs in GSE76855 dataset, 18,115 mRNAs and 1,944 lncRNAs in GSE32323 dataset, 8,455 mRNAs and 597 lncRNAs in GSE62321 dataset, 18,115 mRNAs and 1,944 lncRNAs in GSE8671 dataset, 18,228 mRNAs and 481 lncRNAs in GSE20842 dataset, 18,115 mRNAs and 1,944 lncRNAs in GSE39582 dataset and 18,545 mRNAs and 1,219 lncRNAs in GSE21815 dataset. With the standard of fold change ≥2.0 and p ≤ 0.05, difference analyses were performed in the eight datasets respectively. Then the method of robust rank aggregation (RRA) was used to integrally calculate the differentially expressed lncRNAs and mRNAs of the eight datasets. Finally, nine lncRNAs (including five up-regulated lncRNAs: UCA1, CRNDE, H19, ZFAS1, BLACAT1, and four down-regulated lncRNAs: LINC00675, DPP10-AS1, LOC344887, HAGLR) and 209 mRNAs (including 80 up-regulated mRNAs and 129 down-regulated mRNAs) were identified as the most significantly differential genes. Detailed information was listed in Supplementary Tables S1 and S2.

### Target mRNAs selection and lncRNA-mRNA co-expression network

**Figure 1:**
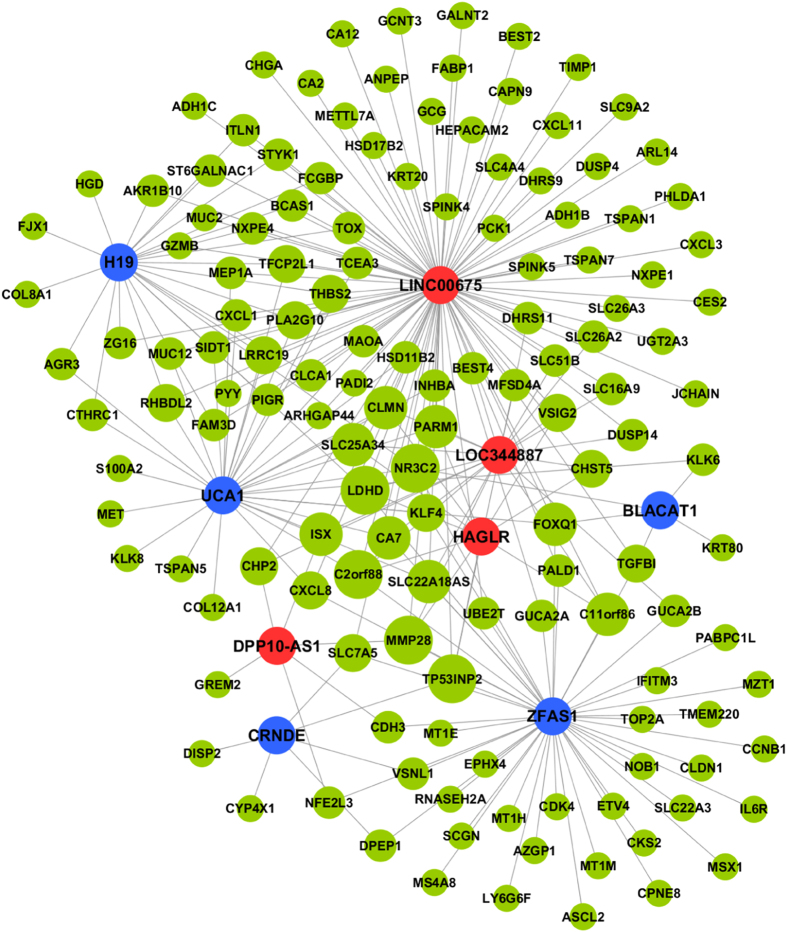
LncRNAs-mRNAs co-expression network. Blue color nodes represent up-regulation, red color nodes represent down-regulation and the others represent mRNA. The size of each node is proportional to the calculated functional connectivity of each lncRNA according to the counts of related target genes.

In order to construct the lncRNA-mRNA co-expression network, we calculated the correlation coefficient between nine lncRNAs and 209 mRNAs selected from microarray data. Eventually, we found 140 mRNAs (48 up-regulated mRNAs and 92 down-regulated mRNAs) had strong correlation with the nine lncRNAs according to the Pearson’s correlation coefficient analysis. Then we constructed the lncRNA-mRNA co-expression network by Cytoscape (version 3.3.0) to visualize the relevance between them (Fig. 1). The size of each node is proportional to the calculated functional connectivity of each lncRNA according to the counts of related target genes. Therefore, significant target genes such as TP53INP2, MMP28, C2orf88, LDHD, and NR3C2 etc. in the network were found to be regulated by multiple lncRNAs. In addition, some related studies show that these genes play a regulatory role in the human body function^17^,^32^,^33^.

### GO and KEGG pathway analyses

**Figure 2:**
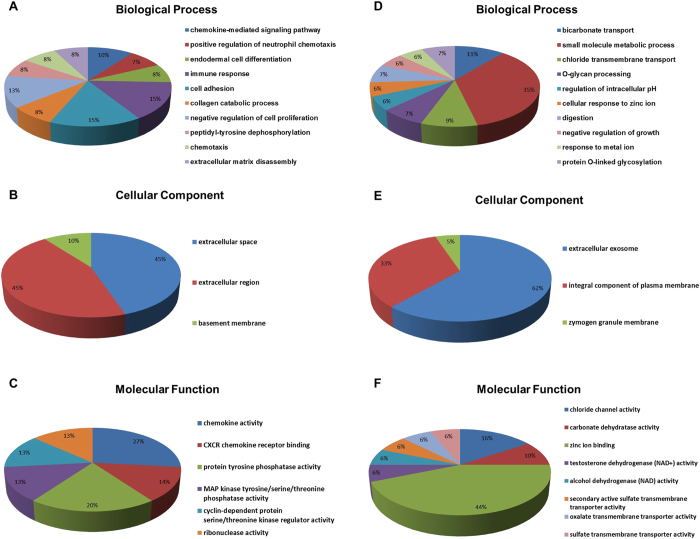
GO terms of the target genes. (**A**) GO analysis of up-regulated target genes according to biological process, (**B**) GO analysis of up-regulated target genes according to cellular component, (**C**) GO analysis of up-regulated target genes according to molecular function, (**D**) GO analysis of down-regulated target genes according to biological process, (**E**) GO analysis of down-regulated target genes according to cellular component, (**F**) GO analysis of down-regulated target genes according to molecular function.

**Figure 3:**
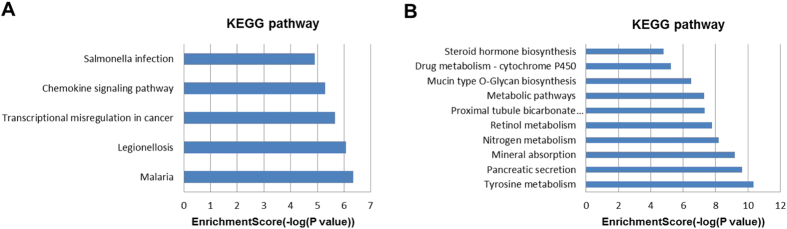
KEGG pathway analysis of target genes. (**A**) Pathways corresponding to the up-regulated target genes. (**B**) Pathways corresponding to the down-regulated target genes.

In our study, the up-regulated and down-regulated mRNAs were performed with Gene ontology (GO) and KEGG pathway analyses separately. In the GO analysis, count ≥2 and p ≤ 0.05 were considered to be statistically significant. Usually, the top 10 objects were selected for concrete analysis. According to the proportion of the count, immune response (15%) and cell adhesion (15%) in the biological process, extracellular space (45%) and extracellular region (45%) in the cellular component, and chemokine activity (27%) in the molecular function were the major terms corresponding to the up-regulated mRNAs, while small molecule metabolic process (35%) in the biological process, extracellular exosome (62%) in the cellular component, and zinc ion binding (44%) in the molecular function were the major terms corresponding to the down-regulated mRNAs (Fig. 2). The p value and other detailed information were listed in Supplementary Table S3. In the KEGG pathway analysis, 5 pathways were associated with the up-regulated mRNAs and 11 pathways related to the down-regulated mRNAs (Fig. 3 and Supplementary Table S4).

### qRT-PCR validation of the nine differentially expressed lncRNAs

#### Validation between CRC tissues and adjacent normal tissues

**Figure 4:**
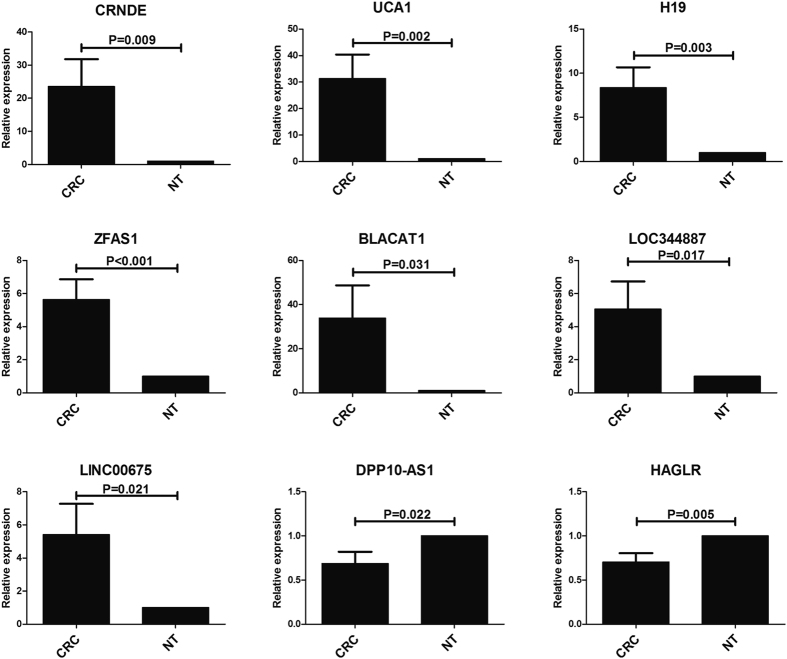
qRT-PCR analysis of nine lncRNAs expression in the CRC tissues and the adjacent non-cancerous tissue. CRC: colorectal cancer; NT, non-cancerous colorectal tissue.

All the nine lncRNAs, five up-regulated ones (UCA1, CRNDE, H19, ZFAS1 and BLACAT1) and four down-regulated ones (LINC00675, DPP10-AS1, LOC344887 and HAGLR), were selected for further verification by qRT-PCR. The expression tendency of the five up-regulated lncRNAs (UCA1, CRNDE, H19, ZFAS1 and BLACAT1) and two down-regulated lncRNAs (DPP10-AS1 and HAGLR) were same as the results of microarray data (p < 0.05). In the two remaining down-regulated lncRNAs (LINC00675 and LOC344887), their expression levels were opposite to the microarray results (p < 0.05). All the detailed information was shown in Fig. 4.

#### Validation in cell lines

To further validate the lncRNA meta-signature, qRT-PCRs were also performed in all three CRC cell lines and normal human colorectal epithelial cell line (FHC). Expression levels of all the lncRNAs were almost consistent with lncRNA microarray results except for the undetectable DPP10-AS1 (Supplementary Figure S1). Moreover, we also detected the supernatant of cell culture, and their expression levels were basically consistent with the results of cells (Supplementary Figure S2).

### Diagnostic value of lncRNA BLACAT1 in serum

**Figure 5:**
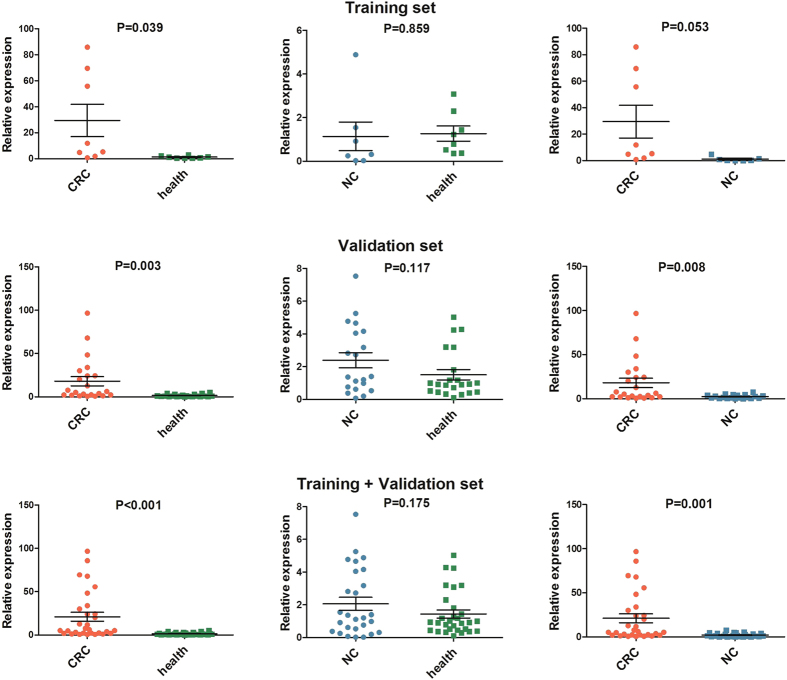
The expression levels of BLACAT1 in serum samples among CRC patients, non-cancer patients and health controls. CRC: colorectal cancer; NC: non-cancer patients.

**Figure 6:**
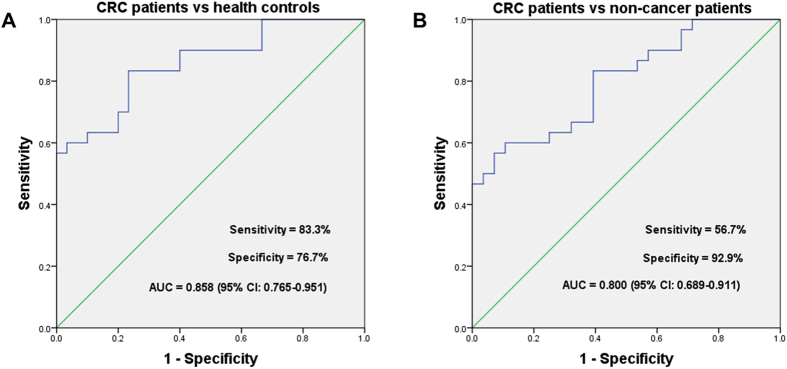
The ROC curve of LncRNA BLACAT1 in the diagnosis of CRC. (**A**) CRC patients vs health control; (**B**) CRC patients vs non-cancer patients.

Since part of lncRNAs were detectable in the supernatant of cell culture, so we established a training set in the serum among CRC patients, non-cancer patients and health controls. The results indicated that only lncRNA ZFAS1 and BLACAT1 were steadily detected among the three groups where they tended to be highly expressed. Then the two screened lncRNAs were further examined in the validation set. The results of the validation set and training + validation set revealed that the expression levels of BLACAT1 in CRC patients were higher than that in health controls (Fig. 5) but ZFAS1 had no significantly statistical difference (Supplementary Figure S3). Meanwhile, it was also found that BLACAT1 was significantly up-regulated between CRC patients and non-cancer patients (Fig. 5). In our study, we also evaluated the diagnostic accuracy of BLACAT1 among CRC patients, non-cancer patients and health controls. The area under the curve (AUC) were 0.858 (95% CI: 0.765–0.951) for BLACAT1 between CRC patients and health controls, and 0.800 (95% CI: 0.689–0.911) between CRC patients and non-cancer patients. The respective sensitivity and specificity were 83.3% and 76.7% for BLACAT1 with the cutoff value of 1.177 between CRC patients and health controls, 56.7% and 92.9% with the cutoff value of 5.035 between CRC patients and non-cancer patients (Fig. 6). The results indicated that lncRNA BLACAT1 was extremely valuable for the diagnosis of CRC.

## Discussion

It has been proven in extensive researches on cancers that the differentially expressed lncRNA pattern was obviously associated with the pathogenesis of various malignant tumors^34^,^35^. In our study, nine lncRNAs meta-signature were incorporated for integrated analysis through a series of data mining and calculation. Among the nine lncRNAs, H19 and UCA1 were the star biomarkers in various types of cancers, such as breast cancer^36^,^37^, gastric cancer^38^,^39^, lung cancer^40^,^41^, and colorectal cancer^42^,^43^. Meanwhile, both of them were closely associated with tumor growth, migration, invasion, proliferation, and prognosis. LncRNA CRNDE was mainly reported to affect the biological characteristics of gliomas^44^,^45^. However, there was only one article about CRC, which reported up-regulated CRNDE as an indicator to poor prognosis^16^. For lncRNA ZFAS1, there were two papers concerning CRC, among which one study reported that up-regulation predicted poor prognosis^46^ while the other illuminated the interaction between ZFAS1 and Cyclin-dependent kinase 1^47^. LncRNA BLACAT1 and four down-regulated lncRNAs (LOC344887, LINC00675, DPP10-AS1, and HAGLR) were all novel biomarkers for the CRC and there were no detailed reports relevant to CRC for now. In recent years, more and more research indicated that mutations and abnormal expression of lncRNAs were closely related to various cancers. Some researchers have also summarized some of the common functions of the lncRNAs, important lncRNA-related diseases, common disease-associated lncRNAs and some classic lncRNA public databases to provide an important reference for clinical disease research^48^. For now, a plenty of lncRNAs have been found, but only a limited number of lncRNAs that is related to diseases can be verified by experiments. Therefore, it has become a heated issue to develop some powerful computational models for lncRNA biomarker identification and prediction such as IRWRLDA^49^, HGIMDA^50^, FMLNCSIM^51^, and LRLSLDA^52^. Based on rapid development of the computer and bioinformatics technology, our study could offer a new perspective and framework for the further research with respect to biological function and serological biomarkers of CRC.

In the present study, we finally identified nine lncRNAs and 140 mRNAs that were differentially expressed between CRC tissues and adjacent normal tissues through a series of integrated analysis. In order to explore the potential function of the differentially expressed transcripts, bioinformatics methods such as GO and KEGG pathway analyses were carried out in our research. A single lncRNA may target multiple transcripts, and a specific transcript may be regulated by multiple lncRNAs, which prompts lncRNAs to induce transformations in various GO processes and signal pathways. In our study, the results of GO analysis indicated that the up-regulated transcripts were principally enriched in cell functions, extracellular component and protease activity, and the down-regulated transcripts were mainly enriched in molecule metabolic processes, exosome and ion activity. Cell function processes consist of cell adhesion, differentiation, proliferation and so on. Dysregulation of all these cellular properties may be closely related to the onset and progression of CRC^53^. Molecule metabolic processes primarily contain small molecule metabolic process, one-carbon metabolic process, xenobiotic metabolic process, which may play an important role in the clinical application in the control, treatment, and prevention of CRC^54^. In short, each process has its value, which may be associated with the occurrence, development and prognosis of CRC.

KEGG pathway analysis demonstrated that 16 pathways were related to the dysregulated transcripts, of which 5 pathways were associated with the up-regulated transcripts and 11 pathways were associated with the down-regulated transcripts. Transcriptional misregulation in cancer and chemokine signaling pathway were the major enriched pathways for up-regulated transcripts, while metabolic pathways and pancreatic secretion were the primary pathways for down-regulated transcripts. These pathways needed to be further studied in that they were highly related to the cancer biology. According to the results of pathways enrichment analyses, we found that the meta-signature lncRNAs were the key regulatory factors for the tumor process and they might be potential therapeutic targets for CRC patients.

After validation between CRC tissues and paired adjacent normal tissues, the results of seven lncRNAs were consistent with the data screened from Gene Expression Omnibus (GEO) database, while the results of two lncRNAs were not. This may attribute to the limitations of data mining algorithm and different test methods mentioned in many related reports^55^,^56^. The qRT-PCR results of cell lines basically corresponded to microarray data. Meanwhile, we found part of the lncRNAs were detectable in the supernatant of cell culture and lncRNAs were reported to be stably expressed in serum samples^57^,^58^. Therefore, in addition to explore the function of target genes of the lncRNAs, we also expanded the scope of our study to find out whether the lncRNAs exhibited differential expression in serum among CRC patients, non-cancer patients and health controls. In this way, some novel biomarkers could be detected as a reference for the diagnosis and differentiation of CRC. In the preliminary serological test, we found two of the nine lncRNAs were detectable by qRT-PCR. Then we increased the sample size and tested the serum of other non-cancer patients. Finally, we found lncRNA BLACAT1 were significant for the diagnosis of CRC. Meanwhile, the BLACAT1 not only has an obviously diagnostic value for CRC, but it has distinctly differential value to separate CRC from other non-cancer diseases. In the co-expression network, we could find that the target genes of lncRNA BLACAT1 were mainly gene NR3C2, FOXQ1, TGFB1, KLK6 and KRT80. Meanwhile, gene NR3C2, FOXQ1 and TGFB1 interacted with multiple genes. It followed that lncRNA BLACAT1 might play an important role in the gene regulation network. However, there are still some limitations in our study, which rest on insufficient sample cases to perform subgroup analysis on clinical characteristics. In the further study, we will increase the sample size and make further study on the cell function and regulatory mechanism of BLACAT1 in CRC.

In summary, nine differentially expressed lncRNAs and 140 mRNAs strongly related to the lncRNAs were identified by data mining. After integrated GO analyses, KEGG signal pathways analyses and a series of experimental validation, we found that the differentially expressed lncRNAs and their potential target mRNAs influenced the pathogenesis of CRC and might be considered as therapeutic targets. Moreover, lncRNA BLACAT1 is found to be at obviously high expression in serum, which might represent a new supplementary biomarker for the diagnosis of CRC.

## Materials and Methods

### CRC gene expression datasets

All the CRC datasets were downloaded from GEO (http://www.ncbi.nlm.nih.gov/geo/). The selection criteria used in this study are as follows: (1) all specimens classified as tissues; cells, serum or plasma are not included; (2) all the included datasets must contain paired CRC tumors and adjacent non-cancerous tissues; (3) both lncRNAs and mRNAs should be included in all the gene expression profiles; (4) sample size should be greater than three pairs; (5) if there existed data overlapping, the largest sample size was selected. According to the above screening criteria, eight datasets were finally included in this study (GSE77199, GSE76855, GSE32323, GSE62321, GSE8671, GSE20842, GSE39582, and GSE21815).

### Microarray data processing and mining

Series matrix files of each dataset were downloaded from GEO. Normalization of the eight datasets was performed by robust multi-array average (RMA) from Gene Expression Console (affymetrix). The normalization procedure such as log2-transformation was separately performed in each gene expression dataset. Then, we screened out the differentially expressed lncRNAs and mRNAs from each dataset on the basis of p ≤ 0.05 and fold change ≥2. Due to the eight datasets from different technological platforms, we could integrally calculate them using the method of RRA, which is suitable and effective for identifying differentially expressed lncRNA meta-signature^59^,^60^. The new data frame results were constructed with the standard of adjusted p value < 0.05. The operation process can be performed by the Robust Rank Aggreg package in R software (version 3.2.3).

### LncRNA functional analyses

#### LncRNA-mRNA co-expression network

The construction of gene co-expression network is mostly developed on basis of the correlation among gene expression profiles. In our study, the lncRNA-mRNA co-expression network construction was mainly divided into three steps: (1) calculate the Pearson’s correlation coefficient between the differentially expressed lncRNAs and mRNAs; (2) identify the differentially expressed mRNAs which significantly correlate with the lncRNAs on basis of the correlation coefficient absolute value of 0.8 (0.8–1.0: extremely strong, 0.6–0.8: strong, 0.4–0.6: moderate, 0.2–0.4: weak, 0–0.2: weak or no correlation); (3) draw the lncRNA-mRNA co-expression network through the software of Cytoscape (version 3.3.0).

#### Gene ontology and pathway analyses

GO, as a representative term to describe the standard features of genes and their products, is widely used in the field of bioinformatics. In this way, the data of gene and gene products can be unified, processed, explained and shared. The relative terms of the GO are divided into three broad categories, including cellular component, molecular function and biological process. According to the GO analysis, we could find the relative terms of the differential gene and further identify differential gene between the samples regarding the change of certain gene function. Pathway analysis is also frequently used in function analysis of the microarray data. However, pathway analysis is different from GO analysis. It employs the resource of biological pathways that have been clearly studied. In our study, the analyses of GO and KEGG pathway were performed with DAVID (Database for Annotation, Visualization, and Integrated Discovery, https://david.ncifcrf.gov/). The selection criteria of the GO terms and KEGG pathways were p ≤ 0.05 and count ≥2.

### Validation of the integrated-signature lncRNAs

#### Tissue samples of CRC patients

To validate the differentially expressed lncRNA-signature, we collected 30 pairs of cancer tissues and adjacent non-cancerous tissues of CRC patients. The patients’ information was summarized in Table 1. There were no statistical difference in gender, age, tumor site and TNM stage. All the tissues were obtained from the pathologically confirmed CRC patients by the experienced surgeons at the Fourth Affiliated Hospital of Guangxi Medical University from 2014 to 2016. Informed consents were obtained from all patients and our experiments were also approved by the Institutional Review Boards of the Fourth Affiliated Hospital of Guangxi Medical University. In addition, all the experiment methods of our study were performed by following with relevant guidelines and regulations. The tissue samples were frozen in liquid nitrogen immediately after surgical resection and subsequently stored at −80 °C before use.

#### Cell culture

FHC and CRC cells (SW480, HT-29 and LS 174T) were obtained from Shanghai Institutes for Biological Sciences. The CRC cells were cultured in Dulbecco’s modified Eagle’s medium (DMEM) or Memorial Institute (RPMI) 1640 medium supplemented with 10% fetal bovine serum (FBS, Sigma Chemical Co., St. Louis, USA) and 100 units/ml antibiotics of penicillin-streptomycin (Invitrogen, Carlsbad, CA) in a humidified incubator with the atmosphere of 5% CO_2_ at 37 °C. FHC were cultured in the base medium of DMEM: F12 medium supplemented with 10 ng/ml cholera toxin, 0.005 mg/ml insulin, 0.005 mg/ml transferrin, 100 ng/ml hydrocortisone, extra 10 mM 4-(2-hydroxyethyl)-1-piperazine-ëthanesulfonic acid (HEPES, for a final concentrate of 25 mM) and 10% FBS (final concentrate). The culture atmosphere and temperature were the same as what was set for the CRC cells. Meanwhile, we also collected the supernatant of cell culture after 24-hour steady growth.

#### Total RNA extraction and quantitative real-time PCR

The total RNA was extracted using the RNAiso Plus (TAKARA BIO INC.) and the concentration was detected by NanoDrop 2000. Total RNA 1 μg was added for synthesizing the first-strand complementary DNA (cDNA) according to the manufacturer’s instructions of the PrimerScript^TM^ RT reagent Kit with gDNA Eraser (TAKARA BIO INC.) The quantitative real-time PCR (qRT-PCR) was performed using the reagent of SYBR Premix Ex Taq^TM^ II (TAKARA BIO INC.) and the 7500 Real-Time PCR Instrument from Life technologies. The primers of all the lncRNAs were obtained from TAKARA and the sequences were listed in Table 2. B2M gene was chosen as an internal control. The PCR thermal cycle process was: 95 °C for 10 min following 40 cycles of 95 °C for 15 s, 60 °C for 1 min, and the melting curve stage was set at the end of the cycles. The relative expression of each lncRNA was calculated on the basis of 2^−ΔΔCt^.

### Serum collection and study design

#### Serum samples of CRC patients, non-cancer patients and health controls

In our study, a total of 90 serum samples were selected for detection, of which 30 samples are collected from CRC patients matched with tissues before surgery, 30 from patients with non-cancer diseases including ulcerative colitis (10 cases), proctitis (10 cases) and intestinal polyp (10 cases), and 30 from sex- and age-matched health controls. All the serum samples were recruited by the Fourth Affiliated Hospital of Guangxi Medical University from 2014 to 2016 and stored at −80 °C before use.

#### Study design

Serum test in our study mainly included three steps: (1) the serum expression levels of the nine lncRNAs were detected in eight CRC patients, eight non-cancer patients and eight health controls to confirm which lncRNAs were really detectable in the serum samples; (2) those detectable lncRNAs in the first step were tested in additional 22 CRC patients, 22 non-cancer patients and 22 health controls; (3) a diagnostic model was constructed to evaluate the diagnostic performance of the differentially expressed lncRNAs in serum samples among the three groups.

## Additional Information

**How to cite this article**: Dai, M. *et al*. Meta-signature LncRNAs serve as novel biomarkers for colorectal cancer: integrated bioinformatics analysis, experimental validation and diagnostic evaluation. *Sci. Rep.* **7**, 46572; doi: 10.1038/srep46572 (2017).

**Publisher's note:** Springer Nature remains neutral with regard to jurisdictional claims in published maps and institutional affiliations.

## Supplementary Material

Supplementary Information

## Figures and Tables

**Table 1:** Clinical characteristics of 30 CRC patients.

Sample NO.	Gender	Age	Tumor site	Tumor morphology	Lymph node	Metastasis	TNM Stage
CRC-1	femal	78	colon	T4	N2	M0	IIIC
CRC-2	femal	54	rectum	T4	N2	M0	IIIC
CRC-3	femal	82	rectum	T4	N1	M0	IIIB
CRC-4	male	62	rectum	T3	N2	M0	IIIC
CRC-5	male	49	colon	T4	N0	M0	IIB
CRC-6	femal	74	rectum	T3	N0	M0	IIA
CRC-7	femal	65	colon	T4	N0	M0	IIB
CRC-8	male	51	rectum	T3	N0	M1	IV
CRC-9	female	73	rectum	T2	N0	M0	I
CRC-10	male	78	colon	T4	N0	M0	IIB
CRC-11	female	24	rectum	T2	N0	M0	I
CRC-12	female	60	colon	T3	N1b	M0	IIIB
CRC-13	male	66	colon	T3	N0	M0	IIA
CRC-14	male	52	colon	T4	N1	M0	IIIB
CRC-15	female	57	colon	T4	N1	M0	IIIB
CRC-16	male	62	rectum	T4	N0	M0	IIB
CRC-17	female	39	colon	T4	N0	M0	IIB
CRC-18	male	63	colon	T3	N1	M0	IIIB
CRC-19	male	55	rectum	T4a	N0	M0	IIB
CRC-20	male	69	colon	T4a	N0	M0	IIB
CRC-21	female	73	colon	T4	N1	M0	IIIB
CRC-22	male	51	colon	T2	N1	M0	IIIA
CRC-23	male	51	colon	T3	N0	M0	IIA
CRC-24	female	47	rectum	T3	N0	M0	IIA
CRC-25	male	87	colon	T4	N2	M1	IV
CRC-26	male	57	colon	T3	N1	M0	IIIB
CRC-27	female	67	colon	T4a	N0	M0	IIB
CRC-28	female	61	rectum	T3	N0	M0	IIA
CRC-29	male	54	rectum	T3	N2	M0	IIIC
CRC-30	male	47	colon	T3	N0	M0	IIA

**Table 2:** The primer sequences of the nine lncRNAs.

Name	Forward	Reverse
CRNDE	5′-ACATGGAAAAATCAAAGTGCTCG-3′	5′-TAACCTTCTTCTGCGTGACAAC-3′
UCA1	5′-CTCTCCATTGGGTTCACCATTC-3′	5′-GCGGCAGGTCTTAAGAGATGAG-3′
H19	5′-CAACATCAAAGACACCATCGG-3′	5′-GAGACAGAAGGATGAAAAAGAAGAA-3′
ZFAS1	5′-GGCTTCATACGCTATTGTCCTG-3′	5′-CTTCCAACACCCGCATTCAT-3′
BLACAT1	5′-ATGACTGACTCCTGACCTTGGCAA-3′	5′-GAGGAAATGGGACTCATCGCC-3′
LOC344887	5′-TCCTCTTTACGGCACAACATTC-3′	5′-TTCCGAGGCTTCTCATTTTACC-3′
LINC00675	5′-ACCACAAGCACCAAAGTCCTAA-3′	5′-GGTCAGTGTCAAAGGGTAGATA-3′
DPP10-AS1	5′-ATCCAGCCCAGATTCTCCTACC-3′	5′-GCCTTCAGGTGGCTGTTTTGTA-3′
HAGLR	5′-GCCTGTGACTGTCCCTTGAATA-3′	5′-GATGTGTGTGGGTGTGTGTTTG-3′
