# Supplementary material for: Meta-signature LncRNAs serve as novel biomarkers for colorectal cancer: integrated bioinformatics analysis, experimental validation and diagnostic evaluation
Source: Sci Rep. 2017 Apr 13;7:46572. doi: 10.1038/srep46572 (PMC5390272; doi:10.1038/srep46572)
Supplement: Supplementary Information [file srep46572-s1.pdf]

Meta-signature LncRNAs serve as novel biomarkers for colorectal cancer: integrated bioinformatics analysis, experimental validation and diagnostic evaluation

Meiyu Dai[1*], Xiaoli Chen[1*], Shanying Mo[1*], Jinwan Li[1], Zhizhuo Huang[1], Shifeng Huang[2], Junyi Xu[2], Baoyu He[1], Yan Zou[1], Jingfan Chen[2$], Shengming Dai[1$]

[1] *Medical Science Laboratory, the Fourth Affiliated Hospital of Guangxi Medical University, Liuzhou, Guangxi 545005, China.*

[2] *Department of General Surgery, the Fourth Affiliated Hospital of Guangxi Medical University, Liuzhou, Guangxi 545005, China.*

[*] Contributed equally to this work.

[$] Corresponding authors

Shengming Dai, No.1 LIUSHI Road, Liuzhou City, Guangxi Province, 545005, China.

Telephone: +86-772-3815334, Fax: +86-772-3837242, E-mail: daishm@sina.com

Jingfan Chen, E-mail: 3203008361@qq.com

This manuscript includes six figures and two tables. Other three figures and four tables were showed in supplementary information.

Supplementary information

Supplementary Table S1. Significantly differentially expressed lncRNAs after integrated calculating through RRA method.

Up-regulated lncRNAs			Down-regulated lncRNAs		
**Name**	**Score**	**Adjusted P value**	**Name**	**Score**	**Adjusted P value**
UCA1	1.94E-16	4.58E-13	LINC00675	1.1E-06	0.002615
CRNDE	5.88E-12	1.39E-08	DPP10-AS1	2.68E-06	0.006349
H19	2.00E-11	4.74E-08	LOC344887	2.99E-06	0.007088
ZFAS1	1.69E-07	0.0004	HAGLR	7.37E-06	0.017434
BLACAT1	3.87E-06	0.009167			

Supplementary Table S2. Significantly differentially-expressed mRNAs after integrated calculating through RRA method.

Up-regulated mRNAs			Down-regulated mRNAs		
**Name**	**Score**	**Adjusted P value**	**Name**	**Score**	**Adjusted P value**
CXCL8	2.11E-16	4.52E-12	CLCA4	2.91E-18	6.25E-14
FOXQ1	3.09E-16	6.62E-12	GUCA2A	7.08E-18	1.52E-13
MMP7	4.61E-15	9.88E-11	CLCA1	1.89E-17	4.05E-13
CDH3	6.92E-15	1.48E-10	ZG16	1.72E-16	3.69E-12
MMP3	6.89E-14	1.48E-09	CA2	1.6E-15	3.43E-11
COL12A1	9.05E-14	1.94E-09	CA4	1.74E-15	3.73E-11
EPHX4	3.13E-13	6.71E-09	CHP2	1.77E-15	3.8E-11
CXCL1	4.75E-13	1.02E-08	ADH1C	2.45E-15	5.25E-11
MMP12	1.90E-12	4.08E-08	VSIG2	3.27E-15	7.01E-11
TGFBI	2.37E-12	5.08E-08	SLC26A2	4.04E-15	8.66E-11
PHLDA1	1.01E-11	2.17E-07	FCGBP	5.61E-15	1.2E-10
CLDN1	3.36E-11	7.21E-07	GCG	1.35E-14	2.9E-10
SLC7A5	4.54E-11	9.73E-07	ITLN1	3.08E-14	6.6E-10
ACSL6	8.42E-11	1.80E-06	PIGR	5.59E-14	1.2E-09
NFE2L3	1.56E-10	3.34E-06	SLC26A3	8.73E-14	1.87E-09
CXCL11	4.75E-10	1.02E-05	SLC51B	1.03E-13	2.2E-09
INHBA	4.95E-10	1.06E-05	GUCA2B	1.39E-13	2.98E-09
FAM3B	8.05E-10	1.73E-05	CHGA	1.86E-13	3.99E-09
CXCL3	1.31E-09	2.80E-05	C2orf88	1.86E-13	3.99E-09
CCNB1	2.24E-09	4.80E-05	MT1M	2.22E-13	4.76E-09
UBE2T	2.67E-09	5.73E-05	HSD17B2	4.24E-13	9.09E-09
S100A2	2.73E-09	5.85E-05	SI	1.03E-12	2.22E-08
APCDD1	2.97E-09	6.38E-05	CA7	2.03E-12	4.35E-08
SRPX2	3.10E-09	6.64E-05	LGALS2	5.23E-12	1.12E-07

STC2	3.69E-09	7.92E-05	BEST2	1.26E-11	2.71E-07
CTHRC1	5.98E-09	0.000128	PCK1	1.53E-11	3.27E-07
IFITM1	6.24E-09	0.000134	INSL5	4.7E-11	1.01E-06
SLCO4A1	6.49E-09	0.000139	SST	6.31E-11	1.35E-06
THBS2	1.33E-08	0.000286	BCAS1	7.24E-11	1.55E-06
GALNT6	1.86E-08	0.000398	LDHD	7.41E-11	1.59E-06
CYP4X1	2.24E-08	0.00048	TSPAN7	9.53E-11	2.04E-06
SLCO1B3	2.25E-08	0.000483	HEPACAM2	1.01E-10	2.15E-06
DUSP14	2.46E-08	0.000527	AGR3	1.11E-10	2.37E-06
CXCL10	2.66E-08	0.000571	AKR1B10	1.14E-10	2.44E-06
CLDN2	2.74E-08	0.000587	MAMDC2	1.7E-10	3.66E-06
SFTA2	3.34E-08	0.000717	TSPAN1	2.55E-10	5.46E-06
GZMB	3.53E-08	0.000756	PYY	3.98E-10	8.53E-06
TPX2	3.81E-08	0.000817	KLF4	5.79E-10	1.24E-05
VSNL1	3.99E-08	0.000855	CD177	6.34E-10	1.36E-05
KLK6	4.67E-08	0.001001	LRRC19	8.99E-10	1.93E-05
TCN1	4.98E-08	0.001068	GREM2	1.03E-09	2.21E-05
AZGP1	9.43E-08	0.002022	PLA2G10	1.25E-09	2.69E-05
CDK4	9.78E-08	0.002098	ANPEP	1.34E-09	2.87E-05
DPEP1	1.00E-07	0.002146	METTL7A	1.44E-09	3.1E-05
ETV4	1.23E-07	0.002644	PARM1	1.66E-09	3.56E-05
COL8A1	1.24E-07	0.002664	HRCT1	1.79E-09	3.83E-05
TOP2A	1.26E-07	0.002705	MT1G	2.05E-09	4.39E-05
IFITM3	1.51E-07	0.003235	NR3C2	2.27E-09	4.87E-05
CKS2	1.58E-07	0.003382	SCGN	2.37E-09	5.07E-05
MZT1	1.69E-07	0.003629	NXPE4	2.5E-09	5.36E-05
SLC22A3	1.88E-07	0.004033	MFSD4A	2.59E-09	5.54E-05
PDCD2L	3.00E-07	0.006426	JCHAIN	2.8E-09	6E-05
KLK8	3.62E-07	0.007764	CA1	2.91E-09	6.23E-05

DDIAS	3.63E-07	0.007783	PADI2	3.31E-09	7.09E-05
ASCL2	3.73E-07	0.008007	SPON1	3.45E-09	7.39E-05
MET	4.10E-07	0.008784	TP53INP2	3.64E-09	7.81E-05
KRT80	4.17E-07	0.00893	TCEA3	3.98E-09	8.53E-05
GPR143	4.22E-07	0.009055	MT1F	4.86E-09	0.000104
DUSP27	4.30E-07	0.009213	BTNL8	5.15E-09	0.00011
IFITM2	4.92E-07	0.010542	UGT2A3	7.24E-09	0.000155
MMP11	5.53E-07	0.011852	FAM3D	7.35E-09	0.000157
DUSP4	5.75E-07	0.012334	CAPN9	8.06E-09	0.000173
MACC1	6.24E-07	0.013374	GALNT2	9.22E-09	0.000198
CHI3L1	6.69E-07	0.01434	MUC12	1.27E-08	0.000272
KRT23	6.74E-07	0.01445	FDCSP	1.31E-08	0.000281
NOB1	7.29E-07	0.015626	BCHE	1.43E-08	0.000307
C2CD4A	8.31E-07	0.017818	ST6GALNAC1	1.57E-08	0.000337
PALD1	9.13E-07	0.019571	NXPE1	1.65E-08	0.000354
MMP1	1.04E-06	0.022225	CA12	1.71E-08	0.000367
RNF183	1.36E-06	0.029263	AQP8	2.31E-08	0.000495
SPARC	1.62E-06	0.034657	MT1H	2.46E-08	0.000528
SPC25	1.66E-06	0.035581	RHBDL2	3.27E-08	0.000702
LY6G6F	1.83E-06	0.03917	FABP1	4.29E-08	0.00092
RNASEH2A	1.85E-06	0.039687	MAOA	4.47E-08	0.000959
MSX1	1.88E-06	0.040406	CES2	4.87E-08	0.001044
PABPC1L	1.89E-06	0.0405	MUC2	5.51E-08	0.001182
TIMP1	1.90E-06	0.04072	DISP2	5.6E-08	0.001201
TSPAN5	2.16E-06	0.046268	LYPD8	6.07E-08	0.001302
REG1A	2.23E-06	0.047826	C11orf86	6.78E-08	0.001455
FJX1	2.28E-06	0.048878	BEST4	7.12E-08	0.001527
			NAP1L2	8.17E-08	0.001752
			SLC9A2	8.27E-08	0.001772

			IL6R	8.28E-08	0.001775
			GCNT3	8.7E-08	0.001865
			CLMN	8.87E-08	0.001902
			TEX11	9.48E-08	0.002032
			MEP1A	1.03E-07	0.002199
			TOX	1.08E-07	0.002326
			ASPA	1.32E-07	0.002822
			TFCP2L1	1.44E-07	0.003095
			DHRS11	1.45E-07	0.003107
			CNTN3	1.61E-07	0.003454
			CLDN8	1.65E-07	0.00354
			SLC4A4	2.04E-07	0.004371
			MS4A12	2.06E-07	0.004421
			TNFRSF17	2.25E-07	0.004832
			SLC16A9	2.25E-07	0.004833
			MS4A8	2.33E-07	0.004995
			CPNE8	2.35E-07	0.005034
			SPINK5	2.55E-07	0.005469
			CHRDL1	2.72E-07	0.005838
			ADAMDEC1	2.93E-07	0.00629
			SLC25A34	2.96E-07	0.006338
			ISX	3.3E-07	0.007067
			ADH1B	3.54E-07	0.00758
			SLC51A	3.87E-07	0.008296
			KRT20	3.94E-07	0.008444
			TMEM220	3.95E-07	0.008461
			GLDN	4.15E-07	0.008892
			STYK1	4.7E-07	0.010077
			PDE6A	4.92E-07	0.010539

			MMP28	5.81E-07	0.012456
			CHST5	6.18E-07	0.013246
			ARHGAP44	6.63E-07	0.01421
			C2orf40	6.72E-07	0.014407
			HSD11B2	6.87E-07	0.014734
			ABCG2	7.99E-07	0.017126
			VIP	9.13E-07	0.019565
			FGL2	1E-06	0.021517
			PLA2G2A	1.15E-06	0.024632
			SIDT1	1.18E-06	0.025336
			ADH1A	1.29E-06	0.027724
			SLC22A18AS	1.42E-06	0.030402
			HGD	1.49E-06	0.031998
			SPINK4	1.66E-06	0.035581
			ARL14	1.7E-06	0.036462
			MT1E	1.74E-06	0.037398
			ANGPTL1	2.01E-06	0.042992
			DHRS9	2.23E-06	0.047826

Supplementary Table S3. Significantly enriched GO processes.

Category and Term	Gene-count	P-value
**Up-regulated mRNAs**		
GO:0070098 chemokine-mediated signaling pathway (BP)	4	0.000889
GO:0090023 positive regulation of neutrophil chemotaxis (BP)	3	0.002119
GO:0035987 endodermal cell differentiation (BP)	3	0.00247
GO:0006955 immune response (BP)	6	0.002475
GO:0007155 cell adhesion (BP)	6	0.005478
GO:0030574 collagen catabolic process (BP)	3	0.01797
GO:0008285 negative regulation of cell proliferation (BP)	5	0.023224
GO:0035335 peptidyl-tyrosine dephosphorylation (BP)	3	0.030704
GO:0006935 chemotaxis (BP)	3	0.042154
GO:0022617 extracellular matrix disassembly (BP)	3	0.042795
GO:0001556 oocyte maturation (BP)	2	0.048245
GO:0005615 extracellular space (CC)	13	4.73E-05
GO:0005576 extracellular region (CC)	13	0.000162
GO:0005604 basement membrane (CC)	3	0.016878
GO:0008009 chemokine activity (MF)	4	0.000283
GO:0045236 CXCR chemokine receptor binding (MF)	2	0.02431
GO:0004725 protein tyrosine phosphatase activity (MF)	3	0.024557
GO:0017017 MAP kinase tyrosine/serine/threonine phosphatase activity (MF)	2	0.034928
GO:0016538 cyclin-dependent protein serine/threonine kinase regulator activity (MF)	2	0.042817
GO:0004540 ribonuclease activity (MF)	2	0.048042
**Down-regulated mRNAs**		
GO:0015701 bicarbonate transport (BP)	6	1.23E-06
GO:0044281 small molecule metabolic process (BP)	19	0.000885
GO:1902476 chloride transmembrane transport (BP)	5	0.000885
GO:0016266 O-glycan processing (BP)	4	0.002641

GO:0051453 regulation of intracellular pH (BP)	3	0.002896
GO:0071294 cellular response to zinc ion (BP)	3	0.002896
GO:0007586 digestion (BP)	4	0.002909
GO:0045926 negative regulation of growth (BP)	3	0.003618
GO:0010038 response to metal ion (BP)	3	0.004416
GO:0006493 protein O-linked glycosylation (BP)	4	0.007224
GO:0006730 one-carbon metabolic process (BP)	3	0.008899
GO:2001225 regulation of chloride transport (BP)	2	0.009483
GO:0006805 xenobiotic metabolic process (BP)	5	0.010236
GO:0055114 oxidation-reduction process (BP)	8	0.018704
GO:0032849 positive regulation of cellular pH reduction (BP)	2	0.018877
GO:0009913 epidermal cell differentiation (BP)	2	0.032804
GO:0006811 ion transport (BP)	4	0.03346
GO:0070062 extracellular exosome (CC)	24	0.008807
GO:0005887 integral component of plasma membrane (CC)	13	0.030713
GO:0042589 zymogen granule membrane (CC)	2	0.044362
GO:0005254 chloride channel activity (MF)	5	0.000128
GO:0004089 carbonate dehydratase activity (MF)	3	0.001842
GO:0008270 zinc ion binding (MF)	14	0.002636
GO:0047035 testosterone dehydrogenase (NAD+) activity (MF)	2	0.027352
GO:0004022 alcohol dehydrogenase (NAD) activity (MF)	2	0.031838
GO:0008271 secondary active sulfate transmembrane transporter activity (MF)	2	0.04958
GO:0019531 oxalate transmembrane transporter activity (MF)	2	0.04958
GO:0015116 sulfate transmembrane transporter activity (MF)	2	0.04958

Supplementary Table S4. Significantly enriched KEGG pathway.

Category and Term	Gene-count	P-value
**Up-regulated mRNAs**		
KEGG:hsa05144 Malaria	3	0.012374
KEGG:hsa05134 Legionellosis	3	0.014899
KEGG:hsa05202 Transcriptional misregulation in cancer	4	0.019781
KEGG:hsa04062 Chemokine signaling pathway	4	0.025456
KEGG:hsa05132 Salmonella infection	3	0.033329
**Down-regulated mRNAs**		
KEGG:hsa00350 Tyrosine metabolism	4	0.000764
KEGG:hsa04972 Pancreatic secretion	5	0.001264
KEGG:hsa04978 Mineral absorption	4	0.001704
KEGG:hsa00910 Nitrogen metabolism	3	0.003435
KEGG:hsa00830 Retinol metabolism	4	0.004581
KEGG:hsa04964 Proximal tubule bicarbonate reclamation	3	0.006266
KEGG:hsa01100 Metabolic pathways	14	0.006342
KEGG:hsa00512 Mucin type O-Glycan biosynthesis	3	0.01122
KEGG:hsa00982 Drug metabolism - cytochrome P450	3	0.02676
KEGG:hsa00140 Steroid hormone biosynthesis	3	0.036532
KEGG:hsa00010 Glycolysis / Gluconeogenesis	3	0.047463

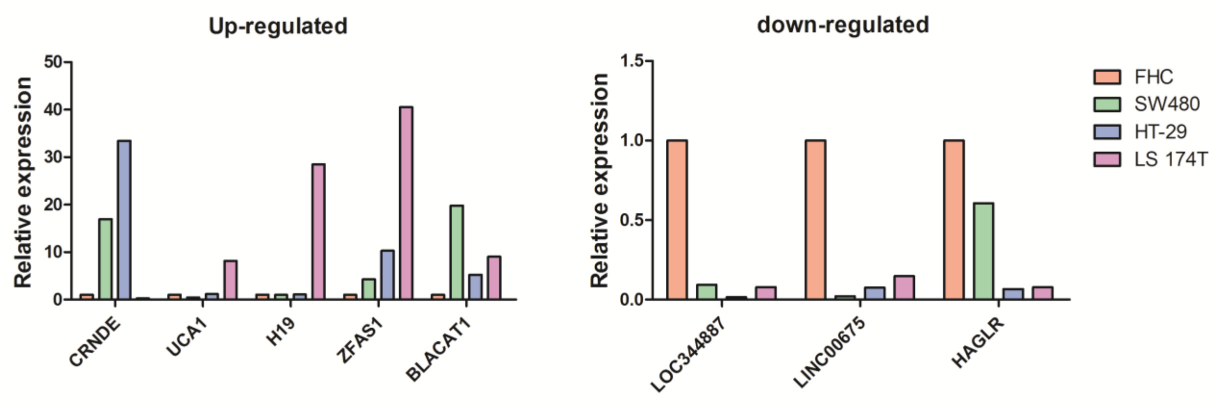

Supplementary Figure S1. qRT-PCR analysis of LncRNAs expression levels in CRC cells and normal cell FHC.

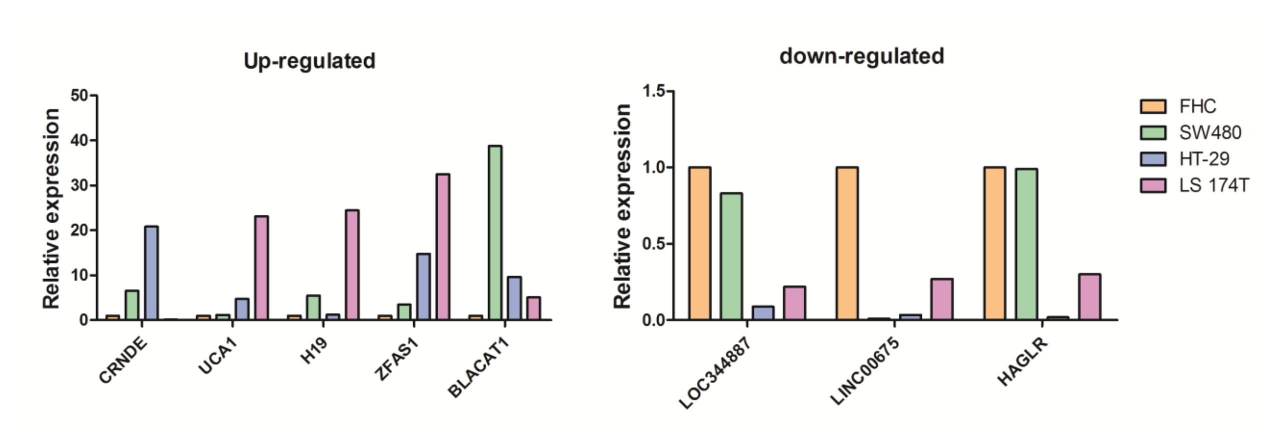

Supplementary Figure S2. qRT-PCR analysis of LncRNAs expression levels in the supernatant of CRC cells and normal cell FHC.

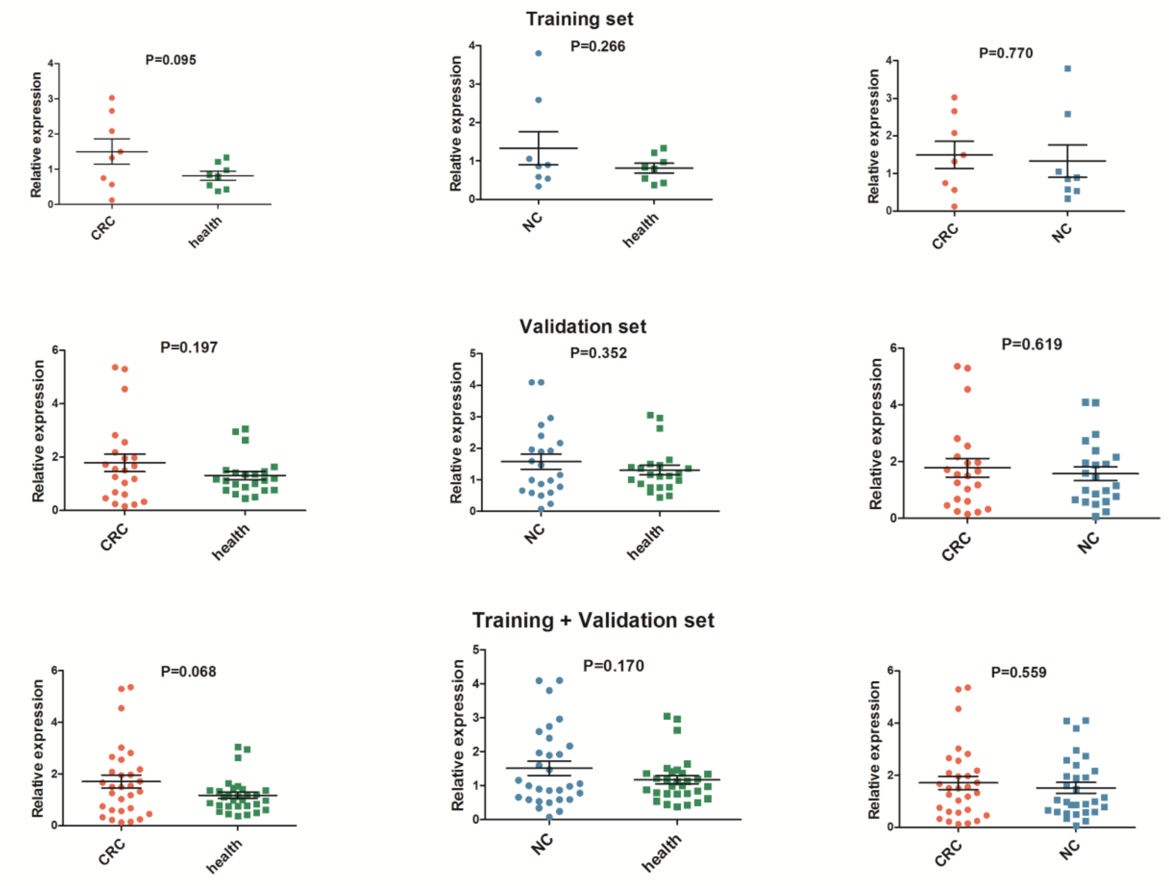

Supplementary Figure S3. The expression levels of ZFAS1 in serum samples among CRC patients, non-cancer patients and health controls. CRC: colorectal cancer; NC: non-cancer patients.
